# Simultaneous preservation of the DNA quality, the community composition and the density of freshwater oligochaetes for the development of genetically based biological indices

**DOI:** 10.7717/peerj.6050

**Published:** 2018-12-05

**Authors:** Régis Vivien, Inge Werner, Benoit J.D. Ferrari

**Affiliations:** Swiss Centre for Applied Ecotoxicology (Ecotox Centre) Eawag-EPFL, Lausanne/Dübendorf, Switzerland

**Keywords:** Aquatic oligochaetes, Bioindication, Fixation, Preservation, Cytochrome c oxidase, PCR amplification, Low-pH formalin, Neutral buffered formalin, Absolute ethanol

## Abstract

**Introduction:**

Oligochaetes are recognized as valuable bioindicators of sediment quality in streams and lakes. The development of an oligochaete index based on the identification of specimens using DNA barcodes requires a method for simultaneously preserving the DNA quality and information on the specimen density and oligochaete community composition. Absolute ethanol optimally preserves DNA but fixation of freshwater oligochaetes with this medium can cause disintegration and fragmentation of specimens. Here, we investigated the possibility to preserve oligochaete specimens in low-pH formalin and in neutral buffered formalin for up to four weeks before genetic analyses and tested if the addition of absolute ethanol to formalin-fixed oligochaetes resulted in a loss of specimens and/or species.

**Methods:**

We performed guanidine extraction and polymerase chain reaction (PCR) amplification/sequencing of a fragment of the cytochrome c oxidase I (COI) gene on tissue fragments preserved in low-pH formalin for up to 3 weeks and in neutral buffered formalin for up to 4 weeks. In addition, we compared the density and taxonomic composition of formalin-fixed oligochaetes of several sieved sediment samples before and after the addition of absolute ethanol.

**Results:**

The COI fragment of all oligochaete specimens preserved in neutral buffered formalin for up to 28 days was successfully amplified by PCR and obtained sequences were complete and of high quality. The amplification success rate for low-pH formalin fixed specimens declined after 7 days of storage. The addition of absolute ethanol to formalin-fixed oligochaete communities did not alter density or diversity estimates.

**Discussion:**

Our results indicate that sediment samples can be stored in neutral buffered formalin for up to 4 weeks and the sieved material can then be transferred to absolute ethanol, without affecting DNA quality, density and community composition of oligochaetes. Based on these results, a protocol for preserving freshwater oligochaetes, describing all the steps from collection of sediments to preservation of the biological material in absolute ethanol, is proposed. This method of fixation/preservation is of relevance for establishing DNA barcode reference databases, inventories of genetic diversity and developing genetically based biological indices.

## Introduction

Freshwater oligochaetes are valuable indicators of sediment quality in rivers and lakes. However, morphological identification to species level is challenging and possible only for a part of the specimens present in the environment (Vivien et al., 2017).

Identifying oligochaete species using DNA barcodes can overcome the issues associated with morphological identification (Vivien et al., 2017). Sanger sequencing of isolated specimens and high throughput sequencing (HTS) of samples composed of genetically tagged specimens (Shokralla et al., 2014) or of pooled specimens (similar quantity of tissue between specimens) constitute possible ways for both identifying the species present in a sample and estimating their abundances. Developing such approaches requires a method for simultaneously preserving the DNA quality, the densities and the community structure of oligochaetes. Absolute ethanol optimally preserves DNA, but the fixation of oligochaetes with this medium can cause disintegration and fragmentation of specimens and so lead to biased abundance and diversity estimates (Rodriguez & Reynoldson, 2011). While formalin is an excellent fixative and optimally preserves oligochaete specimens, this medium is much less appropriate for DNA preservation than absolute ethanol (Timm & Martin, 2015). Formalin induces negative effects on DNA, such as covalent cross linking, irreversible denaturation, modification and fragmentation (Chaw et al., 1980; Tang, 2006; Hykin, Bi & McGuire, 2015). However, two factors influence the success of amplification and sequencing of DNA from formalin fixed tissues: the duration of preservation in formalin and the pH of formalin. The amplification success rate decreases with the increase of preservation time in formalin (Schander & Halanych, 2003; Bucklin & Allen, 2004; Baird et al., 2011). Low-pH formalin causes more degradation of DNA than neutral buffered formalin, especially for long preservation time (Koshiba et al., 1993; Schander & Halanych, 2003).

The simultaneous preservation of oligochaete communities/densities and DNA is possible. Vivien, Ferrari & Pawlowski (2016) showed that a COI fragment (658 pb) of freshwater oligochaete tissues preserved in low-pH formalin for up to one week before their transfer to absolute ethanol (at −20 °C) could be successfully amplified by PCR and sequenced. According to these results, sediment samples can be stored in low-pH formalin for maximum one week before sieving and then the sieved material can be preserved in absolute ethanol at −20 °C. However, this method has an important disadvantage. Indeed, the maximum time of preservation in formalin is quite short. It implies that the sieving of sediments must be done rapidly after sampling, which is not always possible as part of routine analyses, especially if the number of sites is large and if the sampling campaigns must be done in a short period. A storage period of at least 2–3 weeks would be necessary between the sampling and the sieving. In addition, it should be verified that the addition of absolute ethanol to formalin-fixed oligochaete communities does not cause a loss of specimens and species.

With the goal of developing a genetic oligochaete index, we tested the possibility to extract and amplify a fragment (658 pb) of the COI gene of oligochaete tissues preserved in low pH formalin for up to 3 weeks and in neutral buffered formalin for up to 4 weeks before being transferred to absolute ethanol. We compared the amplification success rate from formalin and ethanol preserved tissue fragments and sequenced the COI fragment of several specimens preserved in neutral buffered formalin to verify that after this treatment high quality and full-length sequences were obtained. In addition, to study the eventual effects of adding absolute ethanol to formalin-fixed oligochaete communities, we compared the density and taxonomic composition of formalin-fixed oligochaetes of several sieved sediment samples before and after the addition of absolute ethanol.

## Material and Methods

### Study on oligochaete DNA preservation in low-pH formalin and neutral buffered formalin

#### Sampling and preparation of samples

Sediment samples were collected in 2017 with a shovel in the Sorge River (Switzerland, Canton of Vaud) (Table S1). Sieving was performed the same day as the collection. After sieving, the samples were stored at 4 °C until sorting. Oligochaetes were sorted out from the sieved sediment samples within a maximum of 10 days after sieving. Each live specimen was cut into three to eight fragments of similar size. One fragment was put directly in absolute ethanol, while the other fragments were stored either in 4% of low-pH (pH = 2.8–4) formalin (Thermo Fisher Scientific, Waltham, MA, USA) or in neutral buffered formalin (Richard-Allan Scientific, Neutral Buffered Formalin 10%) at 4 °C for several time periods (3 min, 1 day, 3 days, 6 days, 7 days, 10 days, 14 days, 21 days or 28 days). We tested two different concentrations of neutral buffered formalin (2 and 4% of formaldehyde) to study the potential influence of the formalin concentration on amplification success. To obtain experimental formaldehyde concentrations, neutral buffered formalin sold by Richard-Allan Scientific (San Diego, CA, USA) was diluted with tap water. At the end of each storage period, formalin-fixed tissues were transferred into tap water for a few seconds and then into absolute ethanol. Once in ethanol, tissues were immediately stored at −20 °C until DNA extraction. The anterior part of several specimens was fixed and preserved in low-pH formalin or absolute ethanol for identification by compound microscope.

#### DNA extraction, PCR and sequencing

Total genomic DNA was extracted from tissue samples using the guanidine thiocyanate method described by Tkach & Pawlowski (1999). A fragment of 658 base pairs of the COI gene was amplified using LCO 1490 and HCO 2198 primers (Folmer et al., 1994). Each PCR was performed in a total volume of 20 µl containing 0.6 Unit of Taq polymerase (Roche), 2 µl of the 10X buffer (Roche, Basel, Switzerland) containing 20 mM of MgCl_2_, 0.5 µl of each primer (10 mM each), 0.4 µl of a mix containing 10 mM of each dNTP (Roche) and 0.8 µl of template DNA of undetermined concentration. The PCR process comprised an initial denaturation step at 95 °C for 5 min, followed by 35 cycles of denaturation at 95 °C for 40 s, annealing at 44 °C for 45 s and elongation at 72 °C for 1 min, with a final elongation step at 72 °C for 8 min. The PCR products were then directly and bi-directionally Sanger sequenced on an ABI 3,031 automated sequencer (Applied Biosystems, Foster City, CA, USA) using the same primers as above and following the manufacturer’s protocol. The raw sequence editing and the generation of contiguous sequences were performed using CodonCode Aligner (CodonCode Corporation, Centerville, MA, USA). Multiple sequence alignments were automatically generated using Muscle v3.8.31 (Edgar, 2004) as implemented in Seaview v.4.4.0 (Gouy, Guindon & Gascuel, 2010).

#### Oligochaete identification

Specimens were identified at the family, sub-family or species level, either by stereo microscope/compound microscope analysis or by genetic analysis. For the identification by compound microscope, the anterior parts were mounted between slide and coverslip in a permanent coating solution composed of lactic acid, glycerol and polyvinylic alcohol (Mowiol 4–88).

The genetic identification was performed using a phylogenetic tree with sequences from this study and from our COI database (Vivien et al., 2017). To construct the phylogenetic tree, the neighbour-joining method as implemented in Seaview v.4.4.0 was applied (Gouy, Guindon & Gascuel, 2010), with 1,000 bootstrap replicates. A 10% threshold of COI divergence was proposed by Erséus & Gustafsson (2009), Zhou et al. (2010), Vivien et al. (2017) and Prantoni et al. (2018) to discriminate between oligochaete species. We applied the same threshold, and therefore considered that a COI divergence of <10% between sequences indicated that specimens belonged to the same species. The genetic distances were calculated using the K2P model in MEGA 5.1 (Tamura et al., 2011).

### Study on densities and community composition of formalin-fixed oligochaetes before and after the addition of absolute ethanol

#### Sampling and examination of oligochaete communities and densities

Sampling was performed between 2015 and 2017. Twelve sediment samples for community analyses were obtained from 11 sites distributed among four rivers and one lake as detailed in Table S1. Sediments from the upper 10 cm were sampled using a Surber type net (0.2 mm mesh size) in the rivers or an Ekman type grab sampler in the lake. Three replicates were taken at each site (one sample every 10–20 m), then combined and fixed with low-pH formaldehyde 37% (final concentration of formaldehyde of 4%) in the field. Both oligochaete community composition and oligochaete density were determined in eight samples, and only oligochaete density was determined in four samples.

In the laboratory, sediment samples were sieved through a column of 5 mm and 0.5 mm mesh size sieves. The material retained at 0.5 mm was transferred into a subsampling square box (5 ×  5 cells). The content of randomly selected cells was transferred into a petri dish and examined under a binocular scope. Successive cells were examined until 100 identifiable oligochaetes were obtained. The oligochaete density (*D*) per 0.1 m^2^ was then calculated using the following formula:

\begin{eqnarray*}D=(N\times C\times 0.1)/(c\times X) \end{eqnarray*}

where: *D* corresponds to the number of oligochaetes per 0.1 m^2^; *N* the number of oligochaetes in the *c* cells prospected; *c* the number of prospected cells; *C* the total number of cells of the subsampling box; *X* the sampled area (in m^2^).

For each of eight samples, 100 sorted specimens were mounted between slide and coverslip in a coating solution composed of lactic acid, glycerol and polyvinylic alcohol (Mowiol 4–88). All specimens were identified to the lowest practical level (species if possible).

The material of the subsampling square of each sample (12 samples) was then transferred into a Tupperware box and preserved in 4% formaldehyde at 4 °C. To study the eventual effects of absolute ethanol on densities and community composition of formalin-fixed oligochaetes, we completely removed formalin from each sample, added absolute ethanol in the samples (final concentration of ethanol of 100%) and stored them at −20 °C for 1 to 3 days. The sorting, determination of densities, mounting and identification of oligochaetes were then performed as described above.

#### Statistical analyses

The correlations between oligochaete densities (per 0.1 m^2^) and the percentages of the families/subfamilies that were frequent in our samples (Tubificinae, Tubificinae with hair setae, Tubificinae without hair setae, Naidinae and Lumbriculidae) obtained before and after addition of absolute ethanol were studied by calculating the coefficient of determination R^2^ and by applying the Pearson test. These analyses were performed using the Free Statistics and Forecasting Software (Wessa, 2017).

## Results

### Low-pH formalin and neutral buffered formalin study

A total of 43 specimens were sorted from the different sieved sediment samples. Nineteen were used for the low-pH formalin study and 24 for the neutral buffered formalin study. 10 specimens were preserved in neutral buffered formaldehyde 2% and 14 in neutral buffered formaldehyde 4%.

Out of the 43 analysed specimens, we identified 39 individuals. The specimens of the low-pH formalin study belonged to 4 different taxa (Table S2): Tubificinae sp, Naidinae sp, Lumbriculidae sp and *Stylodrilus heringianus*. Those of the neutral buffered formalin study belonged to 8 different taxa: 3 lineages of *Tubifex tubifex*, 2 lineages of *Limnodrilus hoffmeisteri*, Tubificinae sp, *Limnodrilus udekemianus* and *Limnodrilus claparedianus*.

**Figure 1:**
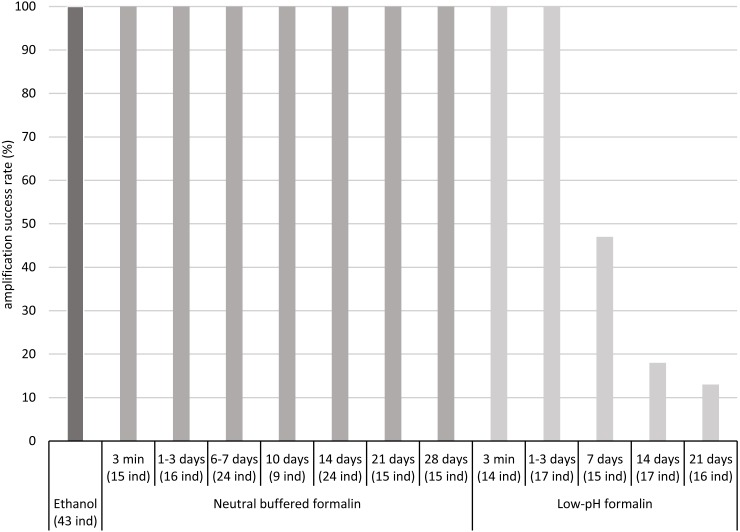
Percentages of amplification success (COI fragment) obtained after fixation/preservation of oligochaete specimens in absolute ethanol and in neutral buffered formalin and low-pH formalin over time. The numbers of analysed specimens for each time and treatment are indicated in brackets (x ind).

The COI fragment of all specimens preserved in low-pH formalin for periods up to 3 days was successfully amplified by PCR, but the COI fragment of only a part of the specimens stored in low-pH formalin for 7 to 21 days was amplified, about 50% after 7 days and less than 20% after 14 and 21 days (Fig. 1, Table S3).

In contrast, the COI fragment of all specimens preserved in neutral buffered formalin for up to 28 days was successfully amplified by PCR. For all samples, intensity of PCR bands was sufficient for Sanger sequencing. We did not observe differences in PCR band intensity between the two different concentrations tested of neutral buffered formalin (2 and 4% of formaldehyde) (Table S3).

Eighteen samples preserved in neutral buffered formalin for periods from 14 days to 28 days (6 for 14 days, 6 for 21 days and 6 for 28 days) were sequenced. All the sequences obtained were whole and of high quality.

**Table 1:** Densities and community compositions of formalin-fixed oligochaetes per site before and after the addition of absolute ethanol. Densities of oligochaetes per 0.1 m^2^, number of oligochaete taxa and percentages of Tubificinae with hair setae, Tubificinae without hair setae, Naidinae, Pristininae, Rhyacodrilinae, Enchytraeidae, Lumbriculidae, Propappidae and Lumbricidae.

Watercourse or lake	Site label	Date of sampling	Before (F) or after (Eth) the addition of absolute ethanol	Density	No of taxa	% Tubificinae with hair setae	% Tubificinae without hair setae	% Naidinae	% Pristininae	% Rhyacodrilinae	% Enchytraeidae	% Lumbriculidae	% Propappidae	% Lumbricidae
canal du Syndicat	site 2	2017	F	24,380	7	25	14	61	0	0	0	0	0	0
canal du Syndicat	site 2	2017	Eth	30,857	10	20	18	61	0	0	1	0	0	0
canal du Bras-Neuf	site 2	2017	F	7,417	9	40	14	46	0	0	0	0	0	0
canal du Bras-Neuf	site 2	2017	Eth	8,667	11	42	9	48	0	0	1	0	0	0
Lake Geneva	site 3	2015	F	2,238	11	83	12	5	0	0	0	0	0	0
Lake Geneva	site 3	2015	Eth	1,705	6	78	19	3	0	0	0	0	0	0
Lake Geneva	site 5	2015	F	1,570	16	77	15	5	0	0	0	3	0	0
Lake Geneva	site 5	2015	Eth	1,646	14	65	29	5	0	0	0	1	0	0
Lake Geneva	site 2	2015	F	479	12	44	35	1	0	0	0	20	0	0
Lake Geneva	site 2	2015	Eth	556	12	48	37	0	0	0	0	15	0	0
Lake Geneva	site 15	2015	F	238	13	21	30	1	0	0	0	48	0	0
Lake Geneva	site 15	2015	Eth	228	9	13	43	0	0	0	0	44	0	0
Ardières	site 3	2016	F	1,063	14	22	62	5	9	0	1	0	1	0
Ardières	site 3	2016	Eth	1,042	13	13	69	2	9	0	3	1	3	0
Ardières	site 3	2017	F	2,292	11	23	42	33	0	1	1	0	0	0
Ardières	site 3	2017	Eth	2,083	11	20	47	30	0	0	2	0	0	1
Venoge	site 1	2015	F	4,074										
Venoge	site 1	2015	Eth	4,622										
Venoge	site 2	2015	F	9,222										
Venoge	site 2	2015	Eth	11,000										
Ardières	site 1	2016	F	1,583										
Ardières	site 1	2016	Eth	2,500										
Ardières	site 2	2017	F	4,583										
Ardières	site 2	2017	Eth	4,500										

### Formalin-fixed oligochaetes before and after the addition of absolute ethanol

The densities and community compositions of formalin-fixed oligochaetes observed before and after the addition of absolute ethanol were close (Table 1, Table S4). The correlation between oligochaete densities determined before and after addition of absolute ethanol was highly significant (*R*^2^ = 0.996, *p* = 1.4∗10^−13^) (Fig. S1). After addition of ethanol, the densities were lower for 5 samples (−1.8% to −24%) and higher for 7 samples (4.6% to 37%). The correlations between the percentages of Tubificinae, Tubificinae with hair setae, Tubificinae without hair setae, Naidinae and Lumbriculidae obtained before and after the addition of absolute ethanol were highly significant (for Tubificinae: *R*^2^ = 0.996, *p* = 2.5*10^−8^; for Tubificinae with hair setae: *R*^2^ = 0.953, *p* = 3.2*10^−5^; for Tubificinae without hair setae: *R*^2^ = 0.906, *p* = 0.0003; for Naidinae: *R*^2^ = 0.996, *p* = 1.8*10^−8^; for Lumbriculidae: *R*^2^ = 0.992, *p* = 1.2*10^−7^) (Fig. S1). The total numbers of taxa (per site) differed before and after the addition of absolute ethanol. After the addition of ethanol, the total numbers of taxa were higher in two samples, lower in four samples and identical in two samples. However, the taxa not recovered or newly found after the addition of ethanol were represented by few specimens (≤6 and mostly only 1 specimen).

## Discussion

Our study has shown that the COI fragment of 658 bp of freshwater oligochaete tissues fixed in neutral buffered formalin and preserved in this medium for up to 4 weeks could be successfully amplified and sequenced. We observed no differences between the amplification success rate of ethanol fixed/preserved samples and neutral buffered formalin fixed/preserved samples. A significant decrease of the amplification success rate of low-pH formalin fixed samples was observed after a storage time of 7 days. The time limit for preservation of tissues in this medium is probably between 3 and 7 days. An amplification success rate close to 100% at a storage time of 7 days, observed in Vivien et al. (2017), was not confirmed in the present work. Our results indicate that the samples should not be stored in low-pH formalin for more than 3 days to guarantee an amplification success rate of 100%. The differences of amplification success observed between neutral buffered formalin and low-pH formalin suggest that the damage of DNA for short time of storage is attributable to the acidity of formalin. Bucklin & Allen (2004) also observed that DNA was more damaged in low-pH formalin than in neutral buffered formalin. These authors showed that neutral buffered formalin was more appropriate for preservation of zooplankton tissues than low-pH formalin, especially if tissues were stored in formalin for a long time. Smaller COI fragments than 658 bp are commonly used for DNA metabarcoding (e.g., Leray et al., 2013). We highlight that the use of neutral buffered formalin for fixation/preservation of oligochaete tissues (for up to 4 weeks) will also be suitable for amplifying such small COI fragments and thus for developing genetic indices based on the analysis of oligochaete samples.

After addition of absolute ethanol to formalin-fixed oligochaete samples, the densities of oligochaetes and the total numbers of taxa did not change significantly. In addition, the percentages of the different families/subfamilies before and after addition of absolute ethanol were similar. We cannot thus conclude that the addition of absolute ethanol induces some loss of specimens and of species. The differences observed before and after the addition of absolute ethanol are probably explained by the natural variability of densities and of composition of taxa between the different cells of the subsampling square. Our results suggest thus that no disintegration or fragmentation of oligochaetes occur when absolute ethanol is added if specimens were beforehand fixed with formalin.

Based on these results, we propose in File S1 a protocol for fixing and preserving oligochaetes, describing the different steps from the collection of sediments to the preservation of the biological material in absolute ethanol. It is important that the sediments are stored in a solution of no more than 4% neutral buffered formaldehyde as the success of amplification and sequencing of the COI fragment from tissues preserved in higher concentrations is not guaranteed. A solution of 4% neutral buffered formaldehyde should be added after having removed the supernatant water (first steps of our protocol).

Our findings make possible to develop a genetic index based on DNA metabarcoding of oligochaete samples. Our method could be applied to other soft bodied organisms, such as leeches, polychaetes and plathelminths which can be also damaged by a direct fixation with absolute ethanol. For morphological investigation of soft bodied invertebrates, formalin is recommended for fixation of organisms and ethanol for long term preservation of the formalin-fixed specimens (Pfannkuche & Thiel, 1988; Mackie, 1994; Wilson, 2005; Häussermann, 2009). However, for the genetic studies of invertebrates, including soft bodied organisms, absolute ethanol is largely used for fixation of organisms (e.g., Krogmann & Holstein, 2010; Elbrecht et al., 2017) as formalin may hamper the subsequent genetic analyses. The use of neutral buffered formalin instead of absolute ethanol for fixing organisms as part of the ecological studies based on HTS analysis of samples composed of sorted invertebrate specimens could constitute a solution for preserving simultaneously the DNA quality, the densities and the community composition of all invertebrates.

## Conclusion

In this study, we have shown that sediment samples could be preserved in neutral buffered formalin (up to 4% of formaldehyde) for up to 4 weeks without affecting oligochaete DNA quality and preventing subsequent PCR amplification of the COI fragment. After sieving, the material can be transferred to absolute ethanol without modifying the density and community composition of oligochaetes. This method of fixation/preservation is relevant for the establishment of comprehensive DNA barcode reference databases and inventories of genetic diversity as well as for the development of new genetically based indices for biomonitoring applications. This method could also prove suitable for simultaneously preserving the DNA quality, the densities and the community composition of other soft bodied invertebrates damageable by a direct fixation with absolute ethanol.

##  Supplemental Information

10.7717/peerj.6050/supp-1 Figure S1 Relationships between the percentages of oligochaete densities and families/subfamilies obtained before and after the addition of absolute ethanol to formalin-fixed oligochaete communities Oligochaete densities per 0.1 m^2^ (A), Tubificinae (B), Tubificinae with hair setae (C), Tubificinae without hair setae (D), Naidinae (E) and Lumbriculidae (F).

10.7717/peerj.6050/supp-2 Table S1 Details about the sampling

10.7717/peerj.6050/supp-3 Table S2 Study on oligochaete DNA preservation in low-pH formalin and neutral buffered formalin: performed analyses per specimen For each sample are indicated the different time periods of preservation in low-pH formalin (from 3 min to 21 days) or in neutral buffered formalin (from 3 min to 28 days), the fixation/preservation in absolute ethanol and the taxonomic identification. X = analysis performed; for samples from 1,121 to 1,129: 2% of formaldehyde; for samples from 1,130 to 1,147: 4% of formaldehyde; Following each taxon name is indicated in brackets how the specimen was identified: 1 = with stereo microscope, 2 = with compound microscope, 3 = with genetic analysis.

10.7717/peerj.6050/supp-4 Table S3 Number of successfully amplified specimens/total number of analysed specimens, for ethanol preservation and low-pH/neutral buffered formalin preservation over time

10.7717/peerj.6050/supp-5 Table S4 Number of specimens of each taxon obtained per site before (F) and after (Eth) the addition of absolute ethanol to formalin-fixed oligochaete communities

10.7717/peerj.6050/supp-6 File S1 Protocol for preserving simultaneously the DNA quality, the densities and the community composition of freshwater oligochaetes

10.7717/peerj.6050/supp-7 File S2 COI sequences of the 18 specimens

## Additional Information and Declarations

### DNA Deposition

The following information was supplied regarding the deposition of DNA sequences: The COI sequences are accessible in the European Nucleotide Archive (ENA) at https://www.ebi.ac.uk/ena/data/view/LR025065-LR025082.

### Data Availability

The following information was supplied regarding data availability: The COI sequences are provided as File S2 and have been deposited in the European Nucleotide Archive (ENA).
