# Supplementary material for: Simultaneous preservation of the DNA quality, the community composition and the density of freshwater oligochaetes for the development of genetically based biological indices
Source: PeerJ. 2018 Dec 5;6:e6050. doi: 10.7717/peerj.6050 (PMC6286655; doi:10.7717/peerj.6050)
Supplement: Figure S1 — Oligochaete densities per 0.1 m2 (A), Tubificinae (B), Tubificinae with hair setae (C), Tubificinae without hair setae (D), Naidinae (E) and Lumbriculidae (F). [file peerj-06-6050-s001.pdf]

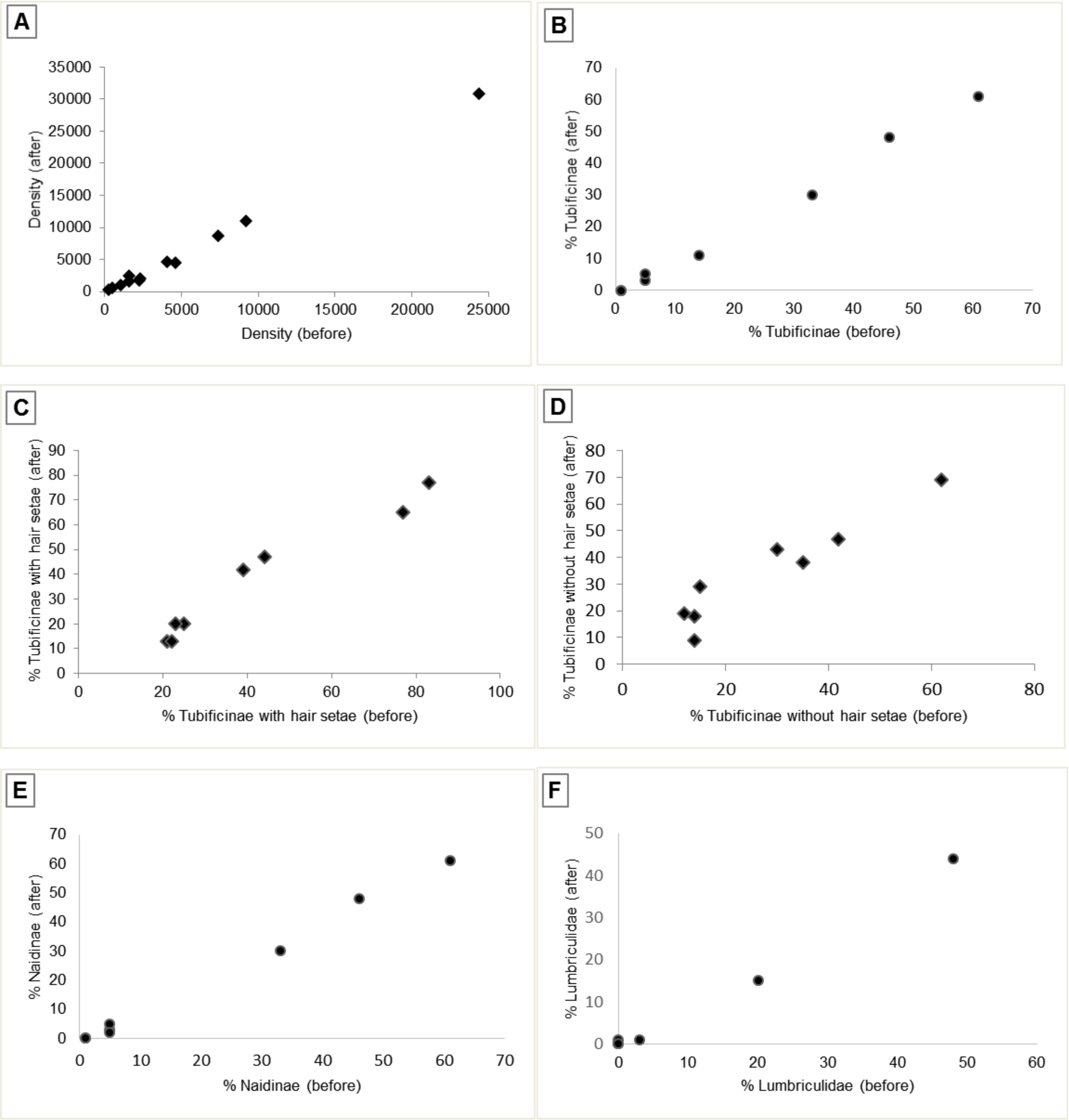

Supplemental Figure S1. Relationships between the percentages of oligochaete densities and families/subfamilies obtained before and after the addition of absolute ethanol to formalin-fixed oligochaete communities. Oligochaete densities per 0.1 m^2 (A), Tubificinae (B), Tubificinae with hair setae (C), Tubificinae without hair setae (D), Naidinae (E) and Lumbriculidae (F)
