# Supplementary material for: Simultaneous preservation of the DNA quality, the community composition and the density of freshwater oligochaetes for the development of genetically based biological indices
Source: PeerJ. 2018 Dec 5;6:e6050. doi: 10.7717/peerj.6050 (PMC6286655; doi:10.7717/peerj.6050)
Supplement: Table S1 [file peerj-06-6050-s002.docx]

Supplemental Table S1. Details about the sampling

| Watercourse | Site label | Country / canton | Sampling year | Coordinates X Y |
| --- | --- | --- | --- | --- |
| Sorge | 1 | Switzerland / Vaud | 2017 | 46.52266^o^N 6.57357^o^E |
| canal du Syndicat | 2 | Switzerland / Valais | 2017 | 46.14060^o^N 7.15459^o^E |
| canal du Bras-Neuf | 2 | Switzerland / Valais | 2017 | 46.28743^o^N 6.94070^o^E |
| Lake Geneva | 3 | Switzerland / Vaud | 2015 | 46.50785^o^N 6.60112^o^E |
| Lake Geneva | 5 | Switzerland / Vaud | 2015 | 46.50577^o^N 6.60090^o^E |
| Lake Geneva | 2 | Switzerland / Vaud | 2015 | 46.50367^o^N 6.60074^o^E |
| Lake Geneva | 15 | Switzerland / Vaud | 2015 | 46.49427^o^N 6.59969^o^E |
| Venoge | 1 | Switzerland / Vaud | 2015 | 46.54270^o^N 6.55138^o^E |
| Venoge | 2 | Switzerland / Vaud | 2015 | 46.54349^o^N 6.55083^o^E |
| Ardières | 1 | France | 2016 | 46.18641^o^N 4.53185^o^E |
| Ardières | 2 | France | 2017 | 46.13275^o^N 4.62476^o^E |
| Ardières | 3 | France | 2016 | 46.12777^o^N 4.71265^o^E |
| Ardières | 3 | France | 2017 | 46.12777^o^N 4.71265^o^E |
