# Supplementary material for: Simultaneous preservation of the DNA quality, the community composition and the density of freshwater oligochaetes for the development of genetically based biological indices
Source: PeerJ. 2018 Dec 5;6:e6050. doi: 10.7717/peerj.6050 (PMC6286655; doi:10.7717/peerj.6050)
Supplement: Table S2 — For each sample are indicated the different time periods of preservation in low-pH formalin (from 3 min to 21 days) or in neutral buffered formalin (from 3 min to 28 days), the fixation/preservation in absolute ethanol and the taxonomic identification. X = analysis performed; for samples from 1,121 to 1,129: 2% of formaldehyde; for samples from 1,130 to 1,147: 4% of formaldehyde; Following each taxon name is indicated in brackets how the specimen was identified: 1 = with stereo microscope, 2 = with compound microscope, 3 = with genetic analysis. [file peerj-06-6050-s003.docx]

Supplemental Table S2. Study on oligochaete DNA preservation in low-pH formalin and neutral buffered formalin: performed analyses per specimen. For each sample are indicated the different time periods of preservation in low-pH formalin (from 3 minutes to 21 days) or in neutral buffered formalin (from 3 minutes to 28 days), the fixation/preservation in absolute ethanol and the taxonomic identification. X = analysis performed; for samples from 1121 to 1129: 2% of formaldehyde; for samples from 1130 to 1147: 4% of formaldehyde; Following each taxon name is indicated in brackets how the specimen was identified: 1 = with stereo microscope, 2 = with compound microscope, 3 = with genetic analysis

| **No sample** | **formalin pH** | **3 min** | **1 d** | **3 d** | **6 d** | **7 d** | **10 d** | **14 d** | **21 d** | **28 d** | **ethanol** | **identification** |
| --- | --- | --- | --- | --- | --- | --- | --- | --- | --- | --- | --- | --- |
| 1049 | low | X |  | X |  | X |  | X | X |  | X | *Stylodrilus heringianus* Claparede, 1862 (1) |
| 1050 | low | X |  | X |  | X |  | X | X |  | X | Tubificinae sp (1) |
| 1051 | low | X |  |  |  | X |  | X | X |  | X | Tubificinae sp (1) |
| 1052 | low |  |  |  |  | X |  | X | X |  | X | Lumbriculidae sp (1) |
| 1053 | low | X |  | X |  | X |  | X | X |  | X | unidentified |
| 1054 | low | X |  | X |  | X |  | X | X |  | X | unidentified |
| 1055 | low | X |  | X |  | X |  | X | X |  | X | unidentified |
| 1056 | low | X |  | X |  | X |  | X | X |  | X | unidentified |
| 1057 | low | X |  | X |  | X |  | X | X |  | X | *Stylodrilus heringianus* Claparede, 1862 (1) |
| 1058 | low | X |  | X |  | X |  | X | X |  | X | Lumbriculidae sp (1) |
| 1059 | low | X |  | X |  | X |  | X | X |  | X | Lumbriculidae sp (1) |
| 1060 | low | X |  | X |  | X |  | X | X |  | X | Lumbriculidae sp (1) |
| 1061 | low | X |  | X |  | X |  | X | X |  | X | Tubificinae sp (1) |
| 1068 | low | X | X |  |  | X |  | X |  |  | X | Lumbriculidae sp (1) |
| 1070 | low |  | X |  |  |  |  | X |  |  | X | Naidinae sp (1) |
| 1071 | low |  | X |  |  |  |  | X |  |  | X | Naidinae sp (1) |
| 1072 | low |  | X |  |  |  |  |  | X |  | X | Naidinae sp (1) |
| 1073 | low |  | X |  |  |  |  |  | X |  | X | Naidinae sp (1) |
| 1074 | low | X | X |  |  | X |  | X | X |  | X | Tubificinae sp (1) |
| 1121 | neutral (2 %) | X |  | X |  | X |  | X | X | X | X | *Tubifex tubifex* (Müller, 1774) (2, 3) |
| 1122 | neutral (2 %) | X |  | X |  | X |  | X | X | X | X | Tubificinae sp (1) |
| 1123 | neutral (2 %) | X |  | X |  | X |  | X | X | X | X | *Limnodrilus hoffmeisteri* Claparede, 1862 (2, 3) |
| 1124 | neutral (2 %) |  |  | X |  | X |  | X | X | X | X | *Tubifex tubifex* (Müller, 1774) (2, 3) |
| 1125 | neutral (2 %) | X |  | X |  | X |  | X | X | X | X | *Tubifex tubifex* (Müller, 1774) (2, 3) |
| 1127 | neutral (2 %) | X |  | X | X | X | X | X |  |  | X | *Tubifex tubifex* (Müller, 1774) (2, 3) |
| 1128 | neutral (2 %) | X |  | X | X | X | X | X |  |  | X | *Tubifex tubifex* (Müller, 1774) (2, 3) |
| 1129 | neutral (2 %) | X |  | X | X | X | X | X |  |  | X | *Tubifex tubifex* (Müller, 1774) (2, 3) |
| 1130 | neutral (4 %) | X |  | X |  | X |  | X | X | X | X | *Tubifex tubifex* (Müller, 1774) (2, 3) |
| 1131 | neutral (4 %) | X |  | X |  | X |  | X | X | X | X | *Tubifex tubifex* (Müller, 1774) (2, 3) |
| 1132 | neutral (4 %) | X |  | X | X | X | X | X |  |  | X | *Tubifex tubifex* (Müller, 1774) (2, 3) |
| 1133 | neutral (4 %) | X |  |  | X | X | X | X |  |  | X | *Tubifex tubifex* (Müller, 1774) (2, 3) |
| 1134 | neutral (4 %) | X |  | X | X | X | X | X |  |  | X | Tubificinae sp (1) |
| 1136 | neutral (2 %) | X |  |  | X | X | X | X |  |  | X | *Tubifex tubifex* (Müller, 1774) (2) |
| 1137 | neutral (2 %) | X |  |  | X | X | X | X |  |  | X | *Limnodrilus hoffmeisteri* Claparede, 1862 (2) |
| 1138 | neutral (4 %) | X |  |  | X | X | X | X |  |  | X | *Tubifex tubifex* (Müller, 1774) (2, 3) |
| 1140 | neutral (4 %) |  |  | X |  | X |  | X | X | X | X | *Limnodrilus udekemianus*  Claparede, 1862 (2) |
| 1141 | neutral (4 %) |  |  | X |  | X |  | X | X | X | X | *Limnodrilus claparedianus* Ratzel, 1868 (2, 3) |
| 1142 | neutral (4 %) |  |  | X |  | X |  | X | X | X | X | *Limnodrilus hoffmeisteri* Claparede, 1862 (2, 3) |
| 1143 | neutral (4 %) |  |  | X |  | X |  | X | X | X | X | *Limnodrilus hoffmeisteri* Claparede, 1862 (2, 3) |
| 1144 | neutral (4 %) |  |  |  |  | X |  | X | X | X | X | *Limnodrilus hoffmeisteri* Claparede, 1862 (2, 3) |
| 1145 | neutral (4 %) |  |  |  |  | X |  | X | X | X | X | *Limnodrilus hoffmeisteri* Claparede, 1862 (2, 3) |
| 1146 | neutral (4 %) |  |  |  |  | X |  | X | X | X | X | Tubificinae sp (1) |
| 1147 | neutral (4 %) |  |  |  |  | X |  | X | X | X | X | *Limnodrilus hoffmeisteri* Claparede, 1862 (2, 3) |
