# Supplementary material for: Simultaneous preservation of the DNA quality, the community composition and the density of freshwater oligochaetes for the development of genetically based biological indices
Source: PeerJ. 2018 Dec 5;6:e6050. doi: 10.7717/peerj.6050 (PMC6286655; doi:10.7717/peerj.6050)
Supplement: Table S3 [file peerj-06-6050-s004.docx]

Supplemental Table S3. Number of successfully amplified specimens / total number of analysed specimens, for ethanol preservation and low-pH / neutral buffered formalin preservation over time.

|  | Storage time in formalin | | | | | | | Ethanol |
| --- | --- | --- | --- | --- | --- | --- | --- | --- |
|  | 3 min | 1-3 days | 6-7 days | 10 d | 14 days | 21 days | 28 days |  |
| Low-pH formalin | 14/14 | 17/17 | 7/15 (7 d) | - | 3/17 | 2/16 | - | 19/19 |
| Neutral buffered formalin (formaldehyde 2%) | 9/9 | 8/8 (3 d) | 10/10 | 5/5 | 10/10 | 5/5 | 5/5 | 10/10 |
| Neutral buffered formalin (formaldehyde 4 %) | 6/6 | 8/8 | 14/14 | 4/4 | 14/14 | 10/10 | 10/10 | 14/14 |
