# Supplementary material for: Simultaneous preservation of the DNA quality, the community composition and the density of freshwater oligochaetes for the development of genetically based biological indices
Source: PeerJ. 2018 Dec 5;6:e6050. doi: 10.7717/peerj.6050 (PMC6286655; doi:10.7717/peerj.6050)
Supplement: Table S4 [file peerj-06-6050-s005.docx]

Supplemental Table S4. Number of specimens of each taxon obtained per site before (F) and after (Eth) the addition of absolute ethanol to formalin-fixed oligochaete communities.

|  | canal du Syndicat site 2 (F) | canal du Syndicat site 2 (Eth) | canal du Bras-Neuf site 2 (F) | canal du Bras-Neuf site 2 (Eth) | Lake Geneva site 3 (F) | Lake Geneva site 3 (Eth) | Lake Geneva site 5 (F) | Lake Geneva site 5 (Eth) | Lake Geneva site 2 (F) | Lake Geneva site 2 (Eth) | Lake Geneva site 15 (F) | Lake Geneva site 15 (Eth) | Ardières site 3 2016 (F) | Ardières site 3 2016 (Eth) | Ardières site 3 2017 (F) | Ardières site 3 2017 (Eth) |
| --- | --- | --- | --- | --- | --- | --- | --- | --- | --- | --- | --- | --- | --- | --- | --- | --- |
| **Tubificinae** |  |  |  |  |  |  |  |  |  |  |  |  |  |  |  |  |
| Tubificinae with hair chaetae | 25 | 19 | 38 | 40 | 43 | 34 | 36 | 39 | 10 | 11 | 11 | 9 | 8 | 4 | 19 | 17 |
| *Tubifex tubifex* (Müller, 1774) |  | 1 | 2 | 2 |  |  | 5 |  | 3 |  | 2 |  |  | 1 |  | 2 |
| *Aulodrilus pluriseta* (Piguet, 1906) |  |  |  |  |  |  | 3 | 2 | 2 | 3 |  |  | 11 | 8 | 4 |  |
| *Psammoryctides barbatus* (Grube, 1861) |  |  |  |  | 4 | 6 |  |  |  |  |  |  | 1 |  |  |  |
| *Embolocephalus velutinus* (Grube, 1879) |  |  |  |  |  |  |  |  | 23 | 22 | 1 |  |  |  |  |  |
| *Potamothrix heuscheri* (Bretscher, 1900) |  |  |  |  |  |  | 1 |  |  | 2 | 1 |  |  |  |  |  |
| *Potamothrix hammoniensis* (Michaelsen, 1901) |  |  |  |  | 2 | 3 | 6 | 3 | 3 | 2 | 2 | 2 |  |  |  |  |
| *Potamothrix vejdovskyi* (Hrabe, 1941) |  |  |  |  |  |  | 13 | 6 | 3 | 8 | 4 | 2 |  |  |  |  |
| *Quistadrilus multisetosus* (Smith, 1900) |  |  |  |  | 32 | 35 | 12 | 15 |  |  |  |  |  |  |  |  |
| *Potamothrix bavaricus* (Oschmann, 1913) |  |  |  |  | 1 |  |  |  |  |  |  |  |  |  |  |  |
| *Lophochaeta ignota* Stolc, 1886 |  |  |  |  | 1 |  | 1 |  |  |  |  |  | 2 |  |  | 1 |
| Tubificinae without hair setae | 8 | 10 | 4 | 5 | 12 | 19 | 13 | 26 | 19 | 24 | 20 | 31 | 55 | 64 | 38 | 45 |
| *Limnodrilus hoffmeisteri* Claparede, 1862 | 6 | 7 | 10 | 4 |  |  |  | 1 | 15 | 5 | 7 | 9 |  |  | 4 | 1 |
| *Limnodrilus claparedianus* Ratzel, 1868 |  |  |  |  |  |  | 1 |  |  | 6 |  | 2 | 7 | 5 |  | 1 |
| *Limnodrilus udekemianus* Claparede, 1862 |  |  |  |  |  |  |  |  |  |  |  |  |  |  |  |  |
| *Limnodrilus profundicola* (Verrill, 1871) |  | 1 |  |  |  |  |  | 1 |  |  |  |  |  |  |  |  |
| *Potamothrix moldaviensis* Vejdovsky & Mrazek, 1903 |  |  |  |  |  |  | 1 | 1 | 1 | 2 | 3 |  |  |  |  |  |
| **Rhyacodrilinae** |  |  |  |  |  |  |  |  |  |  |  |  |  |  |  |  |
| *Bothrioneurum vejdovskyanum* Stolc, 1886 |  |  |  |  |  |  |  |  |  |  |  |  |  |  | 1 |  |
| **Lumbriculidae** |  |  |  |  |  |  |  |  |  |  |  |  |  |  |  |  |
| Lumbriculidae non reconnaissable à l’état immature |  |  |  |  |  |  | 1 |  | 9 | 12 | 26 | 28 |  | 1 |  |  |
| *Stylodrilus heringianus* Claparede, 1862 |  |  |  |  |  |  |  |  | 11 | 3 | 18 | 15 |  |  |  |  |
| *Stylodrilus lemani* (Grube, 1879) |  |  |  |  |  |  |  |  |  |  | 4 | 2 |  |  |  |  |
| *Lumbriculus variegatus* (Muller, 1774) |  |  |  |  |  |  | 2 | 1 |  |  |  |  |  |  |  |  |
| **Naidinae** |  |  |  |  |  |  |  |  |  |  |  |  |  |  |  |  |
| *Ophidonais serpentina* (Müller, 1774) |  |  |  |  |  |  | 1 |  |  |  |  |  |  |  |  |  |
| *Piguetiella blanci* (Piguet, 1906) |  |  |  |  |  |  | 1 | 1 |  |  |  |  |  |  |  |  |
| *Specaria josinae* (Vejdovsky, 1884) |  |  |  |  | 1 |  |  |  |  |  |  |  |  |  |  |  |
| *Uncinais uncinata* (Orsted, 1842) |  |  |  |  | 1 |  | 3 | 1 | 1 |  |  |  |  |  |  |  |
| *Nais pardalis* Piguet, 1906 |  |  |  |  | 1 |  |  |  |  |  |  |  |  |  | 2 |  |
| *Nais communis* Piguet, 1906 | 1 | 3 | 2 | 2 |  |  |  |  |  |  |  |  | 2 | 1 | 2 | 2 |
| *Nais elinguis* Müller, 1774 | 52 | 50 | 32 | 31 |  |  |  |  |  |  |  |  |  |  | 26 | 28 |
| *Nais christinae* Kasprzak, 1973 | 6 | 2 | 2 | 1 |  |  |  |  |  |  |  |  |  |  | 1 |  |
| *Nais barbata* Muller, 1774 | 2 | 6 | 4 | 2 |  |  |  |  |  |  |  |  |  |  |  |  |
| *Dero digitata* (Muller, 1774) |  |  | 6 | 11 |  |  |  |  |  |  |  |  |  |  |  |  |
| *Chaetogaster diaphanus* (Gruithuisen, 1828) |  |  |  | 1 |  | 3 |  | 1 |  |  |  |  |  |  |  |  |
| *Chaetogaster diastrophus* (Gruithuisen, 1828) |  |  |  |  |  |  |  |  |  |  |  |  | 1 |  |  |  |
| *Vejdovskyella comata* (Vejdovsky, 1884) |  |  |  |  |  |  |  |  |  |  |  |  | 1 | 1 |  |  |
| *Vejdovskyella intermedia* (Bretscher, 1896) |  |  |  |  | 2 |  |  | 2 |  |  | 1 |  | 1 |  | 2 |  |
| **Pristininae** |  |  |  |  |  |  |  |  |  |  |  |  |  |  |  |  |
| *Pristina foreli* (Piguet, 1906) |  |  |  |  |  |  |  |  |  |  |  |  | 1 | 1 |  |  |
| *Pristina longiseta* Ehrenberg, 1828 |  |  |  |  |  |  |  |  |  |  |  |  |  | 1 |  |  |
| *Pristina menoni* (Aiyer, 1929) |  |  |  |  |  |  |  |  |  |  |  |  | 8 | 7 |  |  |
| **Enchytraeidae** |  |  |  |  |  |  |  |  |  |  |  |  |  |  |  |  |
| *Globulidrilus riparius* Bretscher, 1899 |  |  |  |  |  |  |  |  |  |  |  |  |  |  | 1 | 2 |
| *Cernosvitoviella* sp |  |  |  |  |  |  |  |  |  |  |  |  | 1 | 3 |  |  |
| *Enchytraeus buchholzi* Vejdovsky, 1878 |  | 1 |  |  |  |  |  |  |  |  |  |  |  |  |  |  |
| *Lumbricillus* sp |  |  |  | 1 |  |  |  |  |  |  |  |  |  |  |  |  |
| **Propappidae** |  |  |  |  |  |  |  |  |  |  |  |  |  |  |  |  |
| *Propappus volki* Michaelsen, 1916 |  |  |  |  |  |  |  |  |  |  |  |  | 1 | 3 |  |  |
| **Lumbricidae** |  |  |  |  |  |  |  |  |  |  |  |  |  |  |  |  |
| *Eiseniella tetraedra* (Savigny, 1826) |  |  |  |  |  |  |  |  |  |  |  |  |  |  |  | 1 |
