# Supplementary material for: Simultaneous preservation of the DNA quality, the community composition and the density of freshwater oligochaetes for the development of genetically based biological indices
Source: PeerJ. 2018 Dec 5;6:e6050. doi: 10.7717/peerj.6050 (PMC6286655; doi:10.7717/peerj.6050)
Supplement: File S1 [file peerj-06-6050-s006.docx]

Supplemental File S1. Protocol for preserving simultaneously the DNA quality, the densities and the community composition of freshwater oligochaetes.

**In the field**

1. Sediment sample is collected in a recipient of 5 litre or 3 litres. Treat the sample immediately after collection

2. Allow particles in suspension to settle for a few seconds/minutes

3. Discard almost all supernatant water, using a sieve of mesh size of 0.16 - 0.2 mm to recuperate eventual worms in suspension. Use the water of the next step for putting back the retained material in the sieve into the recipient.

4. Add 1 litre of neutral buffered formalin 10% (= 4% formaldehyde) in the recipient: first, add 800 ml of river/lake water, and then 200 ml of neutral buffered formalin (20% formaldehyde) (dilution 1/5).

5. Seal the container and mix gently by inversion and rotation to allow distribution of formalin through all the sediment.

6. The container must be preserved at 4^o^C for at least one night to ensure that all the worms present in the sample are fixed. Proceed to the sieving of sediments as soon as possible and maximum four weeks after collection of sediments

**In the laboratory**

The next steps should be performed in a fume hood for avoiding inhalation of formalin vapours.

7. Discard the supernatant (containing about 4% formaldehyde)

8. Sediments are sieved with tap water using a column of two sieves, the first of mesh size of 5 mm (to remove coarse particles) and the second of mesh size of 0.16 mm to 0.5 mm

9. Transfer the material retained in the sieve of 0.16-0.5 mm mesh size to a Tupperware box

10. Remove all the supernatant water of the Tupperware box using a sieve of mesh size of 0.16-0.2 mm

11. Put back the material retained in the sieve into the Tupperware box using some absolute ethanol (100%)

12. Add about 200-300 ml of absolute ethanol in the Tupperware box. Make sure that the final concentration of ethanol in the box is 100% or very close to 100%.

13. Preserve the Tupperware box at -20 ^o^C.
