# Supplementary material for: Simultaneous preservation of the DNA quality, the community composition and the density of freshwater oligochaetes for the development of genetically based biological indices
Source: PeerJ. 2018 Dec 5;6:e6050. doi: 10.7717/peerj.6050 (PMC6286655; doi:10.7717/peerj.6050)
Supplement: File S2 [file peerj-06-6050-s007.docx]

Supplemental File S2. COI sequences (of the 18 specimens)

>1121_Tubifex_tubifex

AACCCTTTATATCGTATTTGGAATTTGAGCTGGAATAGTAGGAACAGGTACAAGCCTCTT

AATTCGCTTAGAATTAGCTCAACCTGGCTCTTTCCTGGGCAGGGACCAACTATATAACAC

TCTAGTTACAGCCCATGCATTCCTGATAATCTTCTTTATAGTAATACCTATCTACATTGG

TGGTTTTGGCAATTGACTGGTTCCACTTATATTAGGGGCACCTGATATAGCATTTCCACG

ATTAAATAACTTAAGATTTTGACTACTACCCCCTTCCTTAATTCTTCTAGTATCTTCTGC

AGCGGTTGAAAAGGGGGCTGGAACTGGGTGAACCGTCTATCCTCCACTATCAAGAAATCT

TGCACACTCGGGCCCATCCGTAGACCTCGCGATCTTCTCACTCCACTTAGCCGGAGTAGC

CTCAATTTTAGGCGCTATCAATTTCATCACCACAATAATTAACATACGATGAAAAGGTAT

GCGGTTAGAACGAATTCCATTATTCGTATGATCAGTAATTCTGACAGTAATTCTATTACT

GCTTACCTTACCTGTACTAGCGGGCGCTATTACTATACTCCTAACAGATCGAAACCTAAA

TACATCATTCTTTGATCCTGCGGGTGGTGGTGATCCAGTTCTCTACCAACATCTATTC

>1123_Limnodrilus_hoffmeisteri

CACCCTATACATAATCTTTGGCCTTTGAGCAGGAATAGTAGGCACAGGAACTAGACTTTT

AATTCGATTTGAGCTAGCACAACCCGGCTCATTTCTCGGTAGAGACCAATTATATAACAC

TCTAGTCACGGCTCACGGATTTTTAATAATTTTCTTTATAGTAATACCTATCTTTATTGG

GGGATTTGGAAATTGATTAGTGCCTTTAATACTTGGAGCACCTGATATGGCATTCCCACG

GCTTAATAACCTAAGATTCTGACTAATGCCCCCATCACTAATTCTACTAGTCTCATCAGC

TGCAGTTGAAAAAGGCGCAGGGACAGGGTGAACTGTATACCCCCCCTTAGCAAGAAATCT

AGCTCATTCCGGGCCTTCTGTAGATCTGGCAATTTTTTCACTACACTTAGCAGGAGCCGC

ATCAATCCTGGGAGCAATTAACTTCATCACAACAATAATCAATATACGATGAAAGGGAAT

ACGCTTAGAGCGTATTCCTTTATTTGTGTGATCCGTAATCATCACAGTTATTTTACTTCT

TCTTACCCTTCCAGTTCTTGCCGGAGCTATCACCATACTTTTAACAGATCGAAACCTAAA

CACCTCATTCTTTGATCCGGCAGGTGGAGGCGATCCAGTTTTATACCAACATCTATTT

>1124_Tubifex_tubifex

AACCCTTTATATCGTATTTGGAATTTGAGCCGGAATAGTAGGAACAGGTACAAGCCTGTT

AATCCGCTTAGAGTTAGCTCAACCTGGCTCTTTCCTGGGCAGGGACCAACTATATAACAC

TCTAGTTACAGCCCATGCATTCCTGATAATCTTCTTTATAGTAATACCTATCTACATTGG

CGGTTTTGGCAATTGACTGGTCCCACTTATATTAGGGGCACCCGATATAGCATTTCCACG

ATTAAATAACTTAAGATTTTGACTACTGCCCCCTTCCTTAATTCTTCTAGTATCTTCTGC

GGCGGTTGAAAAGGGGGCTGGAACAGGATGAACTGTCTATCCTCCACTATCAAGAAACCT

TGCACATTCAGGCCCATCCGTAGATCTCGCAATCTTCTCACTCCACTTAGCCGGGGTGGC

CTCAATTTTAGGTGCTATCAACTTCATCACCACAATAATTAATATACGATGAAAAGGTAT

ACGGTTAGAACGCATTCCGTTGTTCGTGTGATCGGTAATTCTGACAGTAATTCTATTACT

GCTTACCTTACCTGTACTAGCGGGCGCTATTACTATACTCCTTACAGATCGAAATCTAAA

TACATCATTCTTTGATCCTGCGGGT---------------------------------

>1125_Tubifex_tubifex

AACTCTATATATCATTTTCGGGATTTGAGCTGGGATGGTCGGGACAGGAACTAGACTCTT

AATTCGTCTAGAACTGGCTCAACCTGGCTCATTCCTTGGAAGGGATCAGCTATACAACAC

CCTAGTTACAGCACATGCATTTCTGATAATTTTCTTCATGGTAATACCTATCTATATTGG

GGGTTTCGGAAATTGGTTAGTACCCTTAATGCTGGGAGCTCCTGACATAGCATTCCCCCG

ATTAAATAATTTAAGATTTTGGTTATTACCCCCTTCCTTAATCCTCCTTGTATCCTCCGC

GGCCGTAGAAAAGGGGGCTGGAACAGGGTGAACAGTATATCCGCCCCTAGCTAGAAATCT

GGCTCATTCTGGGCCTTCTGTAGACTTAGCTATTTTCTCTCTACATTTAGCTGGGGTGGC

ATCGATTCTAGGAGCTATTAACTTTATTACTACAATAATTAACATGCGATGAAAAGGGAT

ACGCCTTGAACGAATTCCCCTATTTGTATGAGCTGTAATTCTAACCGTAATCTTACTTTT

ACTAACATTACCAGTTTTAGCTGGTGCCATTACCATGCTACTAACAGATCGAAATCTAAA

CACATCCTTCTTTGACCCTGCTGG----------------------------------

>1127_Tubifex_tubifex

GACCCTTTATATCGTATTTGGAATTTGAGCTGGAATAGTAGGAACAGGTACAAGCCTCTT

AATCCGCTTAGAATTAGCTCAACCTGGCTCTTTCCTGGGCAGAGACCAACTATATAACAC

TCTAGTTACAGCCCATGCATTCCTGATAATCTTCTTTATAGTAATACCTATCTACATTGG

TGGTTTTGGCAATTGACTGGTCCCACTTATATTAGGGGCACCCGATATAGCATTTCCACG

ATTAAATAACTTAAGATTTTGACTACTACCCCCTTCCTTAATTCTTCTAGTATCATCTGC

AGCGGTTGAAAAAGGAGCTGGAACTGGGTGAACCGTTTATCCTCCACTATCAAGAAATCT

TGCACACTCGGGCCCATCCGTAGACCTTGCAATCTTCTCACTCCACTTAGCCGGAGTAGC

CTCAATTTTAGGCGCTATCAATTTCATCACCACAATAATTAACATACGATGAAAAGGTAT

ACGGTTAGAACGAATTCCATTATTCGTGTGATCAGTAATTCTGACAGTAATTCTATTACT

GCTTACCTTACCTGTACTAGCAGGCGCTATTACTATACTCCTAACAGATCGAAACCTAAA

TACATCATTCTTTGATCCTGCGGGTGGTGGTGATCCAGTTCTTTACCAACATCTATTC

>1128_Tubifex_tubifex

AACCCTTTATATAGTATTTGGTATTTGAGCTGGTATAGTAGGCACTGGAACAAGTTTATT

AATTCGTTTAGAATTAGCTCAACCTGGCTCCTTCTTAGGCAGAGATCAATTATATAACAC

CTTAGTTACAGCCCACGCCTTCCTGATAATCTTCTTTATGGTAATGCCAATCTACATTGG

TGGCTTCGGAAACTGACTAGTACCACTAATGCTAGGGGCACCAGACATAGCATTCCCCCG

ACTAAATAACCTAAGATTTTGACTATTACCTCCATCCCTAATCTTACTTGTATCATCTGC

TGCAGTAGAAAAAGGGGCAGGAACAGGTTGAACTGTATACCCTCCCCTAGCTAGAAATCT

AGCACATTCCGGACCCTCCGTAGACCTGGCTATCTTCTCACTACATTTAGCTGGTGTAGC

ATCAATTCTAGGAGCCATTAATTTCATTACCACAATAATCAATATACGCTGAAAAGGTAT

ACGCCTAGAACGTATTCCTTTATTCGTATGATCAGTTATTATTACTGTAATCCTTCTATT

ACTCACACTCCCAGTACTAGCCGGTGCTATTACTATACTTCTTACAGACCGAAATCTAAA

CACCTCATTCTTCGACCCTGCTGGTGGTGGAGACCCTGTCCTTTACCAACATCTATTC

>1129_Tubifex_tubifex

AACCCTTTATATCGTATTTGGAATTTGAGCTGGAATAGTAGGAACAGGTACAAGCCTCTT

AATCCGCTTAGAATTAGCTCAACCTGGCTCTTTCCTGGGCAGAGACCAACTATATAACAC

TCTAGTTACAGCCCATGCATTCCTGATAATCTTCTTTATAGTAATACCTATCTACATTGG

TGGTTTTGGCAATTGACTGGTCCCACTTATATTAGGGGCACCCGATATAGCATTTCCACG

ATTAAATAACTTAAGATTTTGACTACTACCCCCTTCCTTAATTCTTCTAGTATCATCTGC

AGCGGTTGAAAAAGGGGCTGGAACTGGGTGAACCGTTTATCCTCCACTATCAAGAAATCT

TGCACACTCGGGCCCATCCGTAGACCTTGCAATCTTCTCACTCCACTTAGCCGGAGTAGC

CTCAATTTTAGGCGCTATCAATTTCATCACCACAATAATTAACATACGATGAAAAGGTAT

ACGGTTAGAACGAATTCCACTATTCGTGTGATCAGTAATTCTGACAGTAATTCTATTACT

GCTTACCTTACCTGTACTAGCAGGCGCTATTACTATACTCCTAACAGATCGAAACCTAAA

TACATCATTCTTTGATCCTGCAGGTGGTGGTGATCCAGTTCTTTACCAACATCTATTC

>1130_Tubifex_tubifex

AACTCTATATATCATTTTCGGGATTTGAGCTGGGATGGTCGGGACAGGAACTAGACTCTT

AATTCGTCTAGAACTGGCTCAACCTGGCTCATTCCTTGGAAGGGATCAGCTATACAACAC

CCTAGTTACAGCACATGCATTTCTGATAATTTTCTTCATGGTAATACCTATCTATATTGG

GGGTTTCGGAAATTGGTTAGTACCCTTAATGCTGGGAGCTCCTGACATAGCATTCCCCCG

ATTAAATAATTTAAGATTTTGGTTATTACCCCCTTCCTTAATCCTCCTTGTATCCTCCGC

GGCCGTAGAAAAGGGGGCTGGAACAGGGTGAACAGTATATCCGCCCCTAGCTAGAAATCT

GGCTCATTCTGGGCCTTCTGTAGACTTAGCTATTTTCTCTCTACATTTAGCTGGGGTGGC

ATCGATTCTAGGAGCTATTAACTTTATTACTACAATAATTAACATGCGATGAAAAGGGAT

ACGCCTTGAACGAATTCCCCTATTTGTATGAGCTGTAATTCTAACCGTAATCTTACTTTT

ACTAACATTACCAGTTTTAGCTGGTGCCATTACCATGCTACTAACAGATCGAAATCTAAA

CACATCCTTCTTTGACCCTGCTGGGGGTGGAGATCCTGTGCTCTATCAACACTTATTC

>1131_Tubifex_tubifex

AACCCTTTATATCGTATTTGGAATTTGAGCTGGGATAGTAGGAACAGGTACAAGCCTCTT

AATCCGCTTAGAATTAGCTCAACCTGGCTCTTTCCTGGGCAGAGACCAACTATATAACAC

TCTAGTTACAGCCCATGCATTCCTGATAATCTTCTTTATAGTAATACCTATCTACATTGG

TGGTTTTGGCAATTGACTGGTCCCACTTATATTAGGGGCACCTGATATAGCATTTCCACG

ATTAAATAACTTAAGATTTTGACTACTACCCCCTTCCTTAATTCTTCTAGTATCATCTGC

AGCGGTTGAAAAAGGGGCTGGAACTGGGTGAACCGTTTATCCTCCACTATCAAGAAATCT

TGCACACTCGGGCCCATCCGTAGACCTTGCAATCTTCTCACTCCACTTAGCCGGAGTAGC

CTCAATTTTAGGCGCTATCAATTTCATCACCACAATAATTAACATACGATGAAAAGGTAT

ACGGTTAGAACGAATTCCATTATTCGTGTGATCAGTAATTCTGACAGTAATTCTATTACT

GCTTACCTTACCTGTACTAGCAGGCGCTATTACTATACTCCTAACAGATCGAAACCTAAA

TACATCATTCTTTGATNCTGCGGGTGGTGGT---------------------------

>1132_Tubifex_tubifex

AACCCTATATATCATTTTCGGGATTTGAGCTGGGATGGTCGGGACAGGAACTAGACTCTT

AATTCGTCTAGAACTGGCTCAACCTGGCTCATTCCTTGGAAGGGATCAGCTATACAACAC

CCTAGTTACAGCACATGCATTTCTGATAATTTTCTTCATGGTAATACCTATCTATATTGG

GGGTTTCGGAAATTGGTTAGTACCCTTAATGCTGGGAGCTCCTGACATAGCATTCCCCCG

ATTAAATAATTTAAGATTTTGGTTATTACCCCCTTCCTTAATCCTCCTTGTATCCTCCGC

GGCCGTAGAAAAGGGGGCTGGAACAGGGTGAACAGTATATCCGCCCCTAGCTAGAAATCT

GGCTCATTCTGGGCCTTCTGTAGACTTAGCTATTTTCTCTCTACATTTAGCTGGGGTGGC

ATCGATTCTAGGAGCTATTAACTTTATTACTACAATAATTAACATGCGATGAAAAGGGAT

ACGCCTTGAACGAATTCCCCTATTTGTATGAGCTGTAATTCTAACCGTAATCTTACTTTT

ACTAACATTACCAGTTTTAGCTGGTGCCATTACCATGCTACTAACAGATCGAAATCTAAA

CACATCCTTCTTTGACCCTGCTGGGGGTGGAGATCCTGTGCTCTATCAACACTTATTC

>1133_Tubifex_tubifex

AACTCTATATATCATTTTCGGGATTTGAGCTGGGATGGTCGGGACAGGAACTAGACTCTT

AATTCGTCTAGAACTGGCTCAACCTGGCTCATTCCTTGGAAGGGATCAGCTATACAACAC

CCTAGTTACAGCACATGCATTTCTGATAATTTTCTTCATGGTAATACCTATCTATATTGG

GGGTTTCGGAAATTGGTTAGTACCCTTAATGCTGGGAGCTCCTGACATAGCATTCCCCCG

ATTAAATAATTTAAGATTTTGGTTATTACCCCCTTCCTTAATCCTCCTTGTATCCTCCGC

GGCCGTAGAAAAGGGGGCTGGAACAGGGTGAACAGTATATCCGCCCCTAGCTAGAAATCT

GGCTCATTCTGGGCCTTCTGTAGACTTAGCTATTTTCTCTCTACATTTAGCTGGGGTGGC

ATCGATTCTAGGAGCTATTAACTTTATTACTACAATAATTAACATGCGATGAAAAGGGAT

ACGCCTTGAACGAATTCCCCTATTTGTATGAGCTGTAATTCTAACCGTAATCTTACTTTT

ACTAACATTACCAGTTTTAGCTGGTGCCATTACCATGCTACTAACAGATCGAAATCTAAA

CACATCCTTCTTTGACCCTGCTGGGGGTGGAGATCCTGTGCTCTATCAACACTTATTC

>1138_Tubifex_tubifex

AACTCTATATATCATTTTCGGGATTTGAGCTGGGATGGTCGGGACAGGAACTAGACTCCT

AATTCGTCTAGAACTGGCTCAACCTGGCTCATTCCTTGGAAGGGATCAGCTATACAACAC

CCTAGTTACAGCACATGCATTTCTGATAATTTTCTTCATGGTAATACCTATCTATATTGG

GGGTTTCGGAAATTGGTTAGTACCCTTAATGCTGGGGGCTCCTGACATAGCATTCCCCCG

ATTAAATAATTTAAGATTCTGGTTATTACCCCCTTCCTTAATCCTCCTTGTATCCTCCGC

GGCCGTAGAAAAGGGGGCTGGAACAGGGTGAACAGTATATCCGCCCCTAGCTAGAAATCT

GGCTCATTCTGGGCCTTCTGTAGACTTAGCTATTTTCTCTCTACATTTAGCTGGGGTGGC

ATCGATTCTAGGAGCTATTAACTTTATTACTACAATAATTAACATACGATGAAAAGGGAT

ACGCCTTGAACGAATTCCCCTATTTGTATGAGCTGTAATTCTAACCGTAATCTTACTTTT

ACTAACATTACCAGTTTTAGCTGGTGCCATTACCATGCTACTAACAGATCGAAATCTAAA

CACATCCTTCTTTGACCCTGCTGGGGGTGGGGATCCTGTGCTCTATCAACACTTATTC

>1141_Limnodrilus_claparedianus

CACTCTCTACATAGTTTTCGGCCTTTGAGCCGGAATAGTCGGTACTGGGACAAGCCTACT

AATTCGATTTGAGCTGGCTCAACCCGGATCATTCTTAGGCAGGGACCAGTTATATAACAC

GTTAGTAACAGCCCACGGATTCTTAATAATTTTCTTTATAGTAATGCCAATCTTTATTGG

TGGGTTTGGAAATTGACTAATTCCCTTAATACTTGGGGCACCAGATATAGCATTCCCACG

ATTAAATAATCTCAGCTTTTGACTAATGCCACCATCATTAATTCTATTAGTGTCATCTGC

TGCTGTAGAAAAAGGTGCCGGCACAGGTTGAACTGTATATCCGCCACTGGCAAGAAATTT

AGCACATTCGGGGCCATCTGTAGATTTAGCAATTTTCTCCCTTCATCTTGCTGGTGCAGC

TTCAATTCTAGGGGCAATTAACTTTATTACAACAATAATTAACATGCGATGAAAAGGAAT

ACGCCTCGAACGAATTCCATTATTCGTATGATCTGTAATTATTACTGTTATTTTACTACT

TCTGACCCTTCCAGTACTTGCGGGGGCTATCACGATACTATTAACAGACCGAAATCTAAA

TACATCGTTCTTCGACCCTGCGGGGGGGGGAGACCCTGTACTTTATCAACACTTATTC

>1142_Limnodrilus_hoffmeisteri

CACCCTATACATAATCTTTGGCCTTTGAGCAGGAATAGTAGGCACAGGAACTAGACTTTT

AATTCGATTTGAGCTAGCACAACCCGGCTCATTTCTCGGTAGAGACCAATTATATAACAC

TCTAGTCACGGCTCACGGATTTTTAATAATTTTCTTTATAGTAATACCTATCTTTATTGG

GGGATTTGGAAATTGATTAGTGCCTTTAATACTTGGAGCACCTGATATGGCATTCCCACG

GCTTAATAACCTAAGATTCTGACTAATGCCCCCATCACTAATTCTACTAGTCTCATCAGC

TGCAGTTGAAAAAGGCGCAGGGACAGGGTGAACTGTATACCCCCCCTTAGCAAGAAATCT

AGCTCATTCCGGGCCTTCTGTAGATCTGGCAATTTTTTCACTACACTTAGCAGGAGCCGC

ATCAATCCTGGGAGCAATTAACTTCATCACAACAATAATCAATATACGATGAAAGGGAAT

ACGCTTAGAGCGTATTCCTTTATTTGTGTGATCCGTAATCATCACAGTTATTTTACTTCT

TCTTACCCTTCCAGTTCTTGCCGGAGCTATCACCATACTTTTAACAGATCGAAACCTAAA

CACCTCATTCTTTGATCCGGCAGGTGGAT-----------------------------

>1143_Limnodrilus_hoffmeisteri

CACCTTATATATAATCTTCGGCCTATGAGCCGGAATAGTGGGCACAGGAACAAGCCTGCT

AATTCGATTCGAATTAGCACAACCTGGTTCATTCCTCGGTAGAGATCAACTCTATAATAC

CTTAGTAACAGCCCACGGTTTCCTTATAATCTTCTTCATGGTAATACCAATTTTTATTGG

TGGCTTCGGAAATTGACTAGTCCCCCTAATGCTAGGAGCTCCTGACATGGCCTTTCCACG

ACTAAATAACCTAAGATTTTGACTAATACCTCCATCACTCATTCTATTAGTTTCATCAGC

CGCGGTCGAAAAGGGAGCGGGGACAGGGTGAACTGTATACCCCCCTCTAGCCAGAAACCT

AGCGCACTCTGGGCCATCTGTGGATCTAGCAATCTTCTCTCTTCACTTAGCCGGGGCTGC

ATCAATTCTAGGTGCCATTAACTTCATTACCACAATAATTAATATACGATGAAAAGGAAT

GCGCCTAGAGCGCATTCCTCTATTTGTATGATCAGTAATCATTACAGTTGTCCTCCTTCT

TCTTACATTACCGGTTTTAGCGGGGGCAATCACCATACTTTTAACAGACCGAAACTTAAA

CACATCATTCTTCGATCCTGCGGGAGGGGGGGACCCGGTACTATATCAACACTTATTT

>1144_Limnodrilus_hoffmeisteri

CACCCTATACATAATCTTTGGCCTTTGAGCAGGAATAGTAGGCACAGGAACTAGACTTTT

AATTCGATTTGAGCTAGCACAACCCGGCTCATTTCTCGGTAGAGACCAATTATATAACAC

TCTAGTCACGGCTCACGGATTTTTAATAATTTTCTTTATAGTAATACCTATCTTTATTGG

GGGATTTGGAAATTGATTAGTGCCTTTAATACTTGGAGCACCTGATATGGCATTCCCACG

GCTTAATAACCTAAGATTCTGACTAATGCCCCCATCACTAATTCTACTAGTCTCATCAGC

TGCAGTTGAAAAAGGCGCAGGGACAGGGTGAACTGTATACCCCCCCTTAGCAAGAAATCT

AGCTCATTCCGGGCCTTCTGTAGATCTGGCAATTTTTTCACTACACTTAGCAGGAGCCGC

ATCAATCCTGGGAGCAATTAACTTCATCACAACAATAATCAATATACGATGAAAGGGAAT

ACGCTTAGAGCGTATTCCTTTATTTGTGTGATCCGTAATCATCACAGTTATTTTACTTCT

TCTTACCCTTCCAGTTCTTGCCGGAGCTATCACCATACTTTTAACAGATCGAAACCTAAA

CACCTCATTCTTTGATCCGGCAGGTGGAGGCGATCCAGTTTTATACCAACATCTATTT

>1145_Limnodrilus_hoffmeisteri

-----------------------------CAGGAATAGTAGGCACAGGAACTAGACTTTT

AATTCGATTTGAGCTAGCACAACCCGGCTCATTTCTCGGTAGAGACCAATTATATAACAC

TCTAGTCACGGCTCACGGATTTTTAATAATTTTCTTTATAGTAATACCTATCTTTATTGG

GGGATTTGGAAATTGATTAGTGCCTTTAATACTTGGAGCACCTGATATGGCATTCCCACG

GCTTAATAACCTAAGATTCTGACTAATGCCCCCATCACTAATTCTACTAGTCTCATCAGC

TGCAGTTGAAAAAGGCGCAGGGACAGGGTGAACTGTATACCCCCCCTTAGCAAGAAATCT

AGCTCATTCNGGGCCTTCTGTAGATCTGGCAATTTTTTCACTACACTTAGCAGGAGCCGC

ATCAATCCTGGGAGCAATTAACTTCATCACAACAATAATCAATATACGATGAAAGGGAAT

ACGCTTAGAGCGTATTCCTTTATTTGTGTGATCCGTAATCATCACAGTTATTTTACTTCT

TCTTACCCTTCCAGTTCTTGCCGGAGCTATCACCATACTTTTAACAGATCGAAACCTAAA

CACCTCATTCTTTGATCCGGCAGGTGGAGGCGATCCAGCTATATACCAACATCTATTT

>1147_Limnodrilus_hoffmeisteri

CACCCTATACATAATCTTTGGCCTTTGAGCAGGAATAGTAGGCACAGGAACTAGACTTTT

AATTCGATTTGAGCTAGCACAACCCGGCTCATTTCTCGGTAGAGACCAATTATATAACAC

TCTAGTCACGGCTCACGGATTTTTAATAATTTTCTTTATAGTAATACCTATCTTTATTGG

GGGATTTGGAAATTGATTAGTGCCTTTAATACTTGGAGCACCTGATATGGCATTCCCACG

GCTTAATAACCTAAGATTCTGACTAATGCCCCCATCACTAATTCTACTAGTCTCATCAGC

TGCAGTTGAAAAAGGCGCAGGGACAGGGTGAACTGTATACCCCCCCTTAGCAAGAAATCT

AGCTCATTCCGGGCCTTCTGTAGATCTGGCAATTTTTTCACTACACTTAGCAGGAGCCGC

ATCAATCCTGGGAGCAATTAACTTCATCACAACAATAATCAATATACGATGAAAGGGAAT

ACGCTTAGAGCGTATTCCTTTATTTGTGTGATCCGTAATCATCACAGTTATTTTACTTCT

TCTTACCCTTCCAGTTCTTGCCGGAGCTATCACCATACTTTTAACAGATCGAAACCTAAA

CACCTCATTCTTTGATCCGGCAGGTGGAGGCGATCCAGTTTTATACCAACATCTATTT
